# Metagenomics as a Transformative Tool for Antibiotic Resistance Surveillance: Highlighting the Impact of Mobile Genetic Elements with a Focus on the Complex Role of Phages

**DOI:** 10.3390/antibiotics14030296

**Published:** 2025-03-12

**Authors:** Nikoline S. Olsen, Leise Riber

**Affiliations:** Department of Plant and Environmental Sciences, University of Copenhagen, Thorvaldsensvej 40, DK-1871 Frederiksberg, Denmark; sno@plen.ku.dk

**Keywords:** antimicrobial resistance (AMR), antibiotic resistance genes (ARGs), surveillance, metagenomics, mobile genetic elements (MGEs), plasmids, bacteriophages (phages), integrons, transposons, phage therapy, horizontal gene transfer (HGT)

## Abstract

Extensive use of antibiotics in human healthcare as well as in agricultural and environmental settings has led to the emergence and spread of antibiotic-resistant bacteria, rendering many infections increasingly difficult to treat. Coupled with the limited development of new antibiotics, the rise of antimicrobial resistance (AMR) has caused a major health crisis worldwide, which calls for immediate action. Strengthening AMR surveillance systems is, therefore, crucial to global and national efforts in combating this escalating threat. This review explores the potential of metagenomics, a sequenced-based approach to analyze entire microbial communities without the need for cultivation, as a transformative and rapid tool for improving AMR surveillance strategies as compared to traditional cultivation-based methods. We emphasize the importance of monitoring mobile genetic elements (MGEs), such as integrons, transposons, plasmids, and bacteriophages (phages), in relation to their critical role in facilitating the dissemination of genetic resistance determinants via horizontal gene transfer (HGT) across diverse environments and clinical settings. In this context, the strengths and limitations of current bioinformatic tools designed to detect AMR-associated MGEs in metagenomic datasets, including the emerging potential of predictive machine learning models, are evaluated. Moreover, the controversial role of phages in AMR transmission is discussed alongside the potential of phage therapy as a promising alternative to conventional antibiotic treatment.

## 1. Introduction

For much of human history, infectious diseases caused by microorganisms have been a leading cause of mortality and healthcare implications [1,2]. The introduction of antibiotics revolutionized medicine, saving millions of lives each year [3,4]. However, the widespread use of antibiotics in both human healthcare and the environment, including agricultural practices, animal husbandry, soil, and water, has led to the emergence and spread of antimicrobial-resistant bacteria, rendering many bacterial infections difficult to treat [5,6,7]. It is estimated that antimicrobial resistance (AMR) was directly responsible for 1.27 million deaths worldwide in 2019 and contributed to an additional of 4.95 million deaths [8]. Hence, AMR is considered to be one of the growing causes of death worldwide, which emphasizes its status as a critical public health priority [8,9].

When exposed to non-lethal doses of antibiotics, a selection pressure is created causing some bacteria to generate adaptive mutations in various chromosomal genes leading to AMR phenotypes insensitive to treatment [10,11]. If recombined onto mobile genetic elements (MGEs), such as integrons, transposons, plasmids, and bacteriophages (phages), these mutations can be further amplified and spread through horizontal gene transfer (HGT), a phenomenon that allows the exchange of genetic material between different bacterial strains by the use of MGEs [12,13,14]. As a result, increasing numbers of pathogenic bacteria accumulate resistance traits to multiple classes of antibiotics and become multidrug-resistant (MDR), extensively drug-resistant (XDR), or even pandrug-resistant (PDR) [15], which poses a serious threat to the effectiveness of antibiotics for treating infections and minor injuries [5,16]. As such, once-treatable infections can become life-threatening, especially for vulnerable populations such as the elderly, immunocompromised individuals, and patients undergoing surgeries or cancer treatments. Consequently, the World Health Organization (WHO) views AMR as one of the top ten threats to global health [17]. This growing threat of AMR calls for immediate action to safeguard public health and to ensure the continued efficacy of antibiotics. Thereby, the need for strengthening surveillance systems to efficiently monitor and control AMR is reinforced [8,9,18].

Robust AMR surveillance, in terms of collection, analysis, and communication of data on resistance patterns, helps track the occurrence, spread, evolution, and impact of resistant pathogens, which not only allows for timely interventions to prevent outbreaks but guides public health strategies, informs appropriate antibiotic use policies, and supports the development of new treatments [19,20]. Additionally, preserving the efficacy of last-resort antibiotics is crucial for managing severe infections, and ongoing monitoring of antibiotic use helps identify when these critical drugs are at risk of becoming ineffective [21,22]. Beyond human health, AMR spreads through agricultural and environmental pathways, emphasizing the importance of the “One Health” approach. This coordinated global framework, formally established in 2009, acknowledges that the health of humans, domestic and wild animals, plants, and the wider environment are closely linked and hence aims to integrate surveillance data from healthcare settings, food production, and the animal and environmental sectors to address AMR holistically [23,24]. Another integrative AMR surveillance strategy, the Global Antimicrobial Resistance and Use Surveillance System (GLASS), was launched by the WHO in 2015 to monitor the status of existing and new national surveillance systems, fill knowledge gaps, and inform strategies at all levels by incorporating data from the One Health sectors on AMR surveillance and consumption of antimicrobials across countries, territories, and areas [25,26].

Here, we address the potential of metagenomics, supported by recent bioinformatic tools such as machine and deep learning models, to improve surveillance strategies for tracking the occurrence and spread of AMR, thereby providing more informed public health decisions as compared to traditional approaches. A particular focus is on the importance of monitoring MGEs in relation to their critical role in facilitating the dissemination of genetic resistance determinants across diverse environmental settings. Moreover, the controversial role of phages as mediators of AMR spread is discussed along with the potential application of phage therapy as a promising and emerging alternative to conventional antibiotic treatments.

## 2. Traditional Tools Used for AMR Surveillance

Historically, AMR surveillance has relied on conventional microbiological and/or molecular techniques to detect and monitor resistant microorganisms. Traditional laboratory-based methods involve cultivating and isolating bacteria from clinical or environmental samples on selective growth media to identify specific pathogens [27]. Once isolated, antimicrobial susceptibility testing (AST) is performed to determine the resistance profile of the isolated bacteria [28].

Common phenotypic AST methods include disk diffusion (Kirby–Bauer test [29]) and broth microdilution, both used to determine if a bacterium is susceptible or resistant to a particular antibiotic. Typically, these culture-based techniques are highly specific and provide quantitative data on resistance patterns, such as minimum inhibitory concentrations (MICs) [30], but they are also time-consuming, hence delaying diagnosis and treatment [31].

To address this, many laboratories use automated systems such as the VITEK^®^ 2 (bioMérieux, Marcy-l’Étoile, France), the PHOENIX Automated Microbiology System (BD Diagnostics, Franklin Lakes, NJ, USA), or MicroScan WalkAway (Beckman Coulter, Brea, CA, USA), which employ optical sensors and databases to rapidly assess bacterial growth in the presence of antibiotics. These systems significantly speed up AST and are widely implemented in clinical laboratories for streamlined AMR detection [32]. Future systems will likely also rely on deep learning-based analysis, e.g., to detect real-time changes in cellular structures, significantly speeding up AST to as little as 30 min [33]. However, results from phenotypic AST assays are not always transferable to treatment outcomes. Complexity arriving from the infection site, potential polymicrobial infections, comorbidity, and the use of non-standardized dosages or multiple drugs can make treatments with antibiotics differ drastically from in vitro assays, and susceptibility predictions can hence be misleading [34].

Today, many national and international AMR surveillance programs, such as the CDC’s National Antimicrobial Resistance Monitoring System (NARMS) [35] or the European Antimicrobial Resistance Surveillance Network (EARS-Net) [36], typically rely on phenotypic AST data collected from hospitals and laboratories to track resistance trends. These programs provide critical insights into the prevalence and distribution of resistance across regions and populations.

Along with traditional AST methods, rapid and efficient molecular tools have been developed to study AMR characteristics. While phenotypic characterization relies on culture-based techniques, excluding many pathogens and microbial communities from detection [27], genotypic approaches like polymerase chain reaction (PCR) and DNA microarrays offer alternative detection strategies. PCR has been widely used in AMR surveillance to identify specific antimicrobial resistance genes (ARGs), such as *blaCTX-M* for extended-spectrum beta-lactamase (ESBL) production [37,38] or *mecA* for methicillin resistance in *Staphylococcus aureus* [39,40]. PCR-based methods are faster than current culture-based approaches, often delivering results within hours. Multiplex PCR further allows the simultaneous detection of multiple ARGs in a single reaction, making it a powerful tool for identifying MDR microorganisms. However, while PCR is highly sensitive and specific for known ARGs, it cannot detect novel or emerging resistance mechanisms unless specific primers for those genes are designed [41]. Also, it provides no information about whether the gene is being actively expressed or contributing to resistance in vivo.

DNA microarrays are used to screen bacterial genomes for known ARGs [42]. Microarrays consist of thousands of probes that hybridize with complementary DNA sequences, producing detectable signals when present. This allows for the simultaneous detection of a wide range of ARGs in a single experiment. However, like PCR, microarrays are limited to detecting genes for which probes are designed and may miss novel or unexpected ARGs. Additionally, microarray data are typically qualitative rather than quantitative, providing information on the presence or absence of genes but not the extent of resistance [43].

Finally, traditional AMR surveillance has often relied on point prevalence surveys, where hospitals, as part of their infection control and public health responsibilities, collect and report AMR data over a specific period to track the proportion of resistant infections [44,45]. This method helps to monitor the geographic spread and prevalence of resistant pathogens within clinical settings and to assess resistance trends across regions and over time. On a global scale, organizations such as the World Health Organization’s GLASS, the European Centre for Disease Prevention and Control (ECDC), and the U.S. National Healthcare Safety Network (NHSN) gather standardized resistance data from clinical laboratories worldwide. However, these networks primarily rely on traditional techniques like cultivation, AST, and PCR, which often focus on a limited number of bacterial species or known ARGs. As a result, they lack the broader picture of microbial communities and might not capture emerging resistance mechanisms. Additionally, data collection is uneven across regions, with low- and middle-income countries often lacking the resources for consistent and comprehensive AMR tracking and surveillance [46].

## 3. Metagenomics as a Transformative Tool for AMR Surveillance

While traditional methods have laid the groundwork for AMR monitoring efforts, they tend to suffer certain limitations in speed and resolution, typically leading to an incomplete picture of the occurrence of both existing and emerging resistance patterns. Hence, they are increasingly complemented by newer techniques, such as metagenomics that define the study of genetic material recovered directly from clinical or environmental samples. Metagenomics enables sequenced-based analysis of entire microbial communities without the need for isolation and laboratory cultivation, and as such offers more comprehensive and rapid insights into AMR dynamics [47,48,49].

The terms metagenome sequencing or metagenomics have been used to describe both (short- and long-read) community shotgun sequencing and community amplicon sequencing [50]. In brief, shotgun sequencing involves random fragmentation and/or ligation, sequencing, assembly, and annotation of the total genomic DNA from a given sample with the aim to identify the entire genetic content of the sequenced microorganisms [51]. Shotgun sequencing, therefore, provides a broad and untargeted view of taxonomic diversity and functional elements, including metabolic pathways, virulence factors, ARGs, and MGEs [51,52,53,54]. Limitations include sequencing- and isolation-related representation bias and high costs [55]. In contrast, amplicon sequencing is a PCR-based method that uses primers to target specific genes like ARGs (AMR-seq) or the 16S rRNA gene as phylogenetic marker in a given sample [56,57]. Amplicon sequencing offers a more targeted and cost-efficient method to determine gene presence or taxonomic composition and often covers a broader diversity as compared to shotgun sequencing but with limited functional insights and only including microorganisms with target sequences [58,59].

Next-generation sequencing (NGS) platforms have significantly advanced both approaches, with second- and third-generation technologies playing major roles, offering complementary strengths. Second-generation technologies, such as Illumina and Ion Torrent, are highly accurate and cost-effective, using massive parallel amplification to sequence short DNA fragments (100–300 bp) [60,61]. The short reads produced by these platforms are ideal for identifying distinct and/or conserved genomic regions, which makes them particularly well suited for amplicon sequencing and for the characterization of ARGs (as in AMR-seq), single-nucleotide polymorphisms (SNPs), and microbial community compositions with precise taxonomic profiling [61,62]. However, their reliance on short reads limits their ability to link ARGs to MGEs and to resolve genomic structures like repetitive sequence elements and the distinction between chromosomal and plasmid contigs, as well as assembling and separating related genomes in complex samples [60,63,64].

In contrast, third-generation single-molecule sequencing platforms like Pacific Biosciences (PacBio) and Oxford Nanopore Technologies (ONT) produce reads with an average length of 1–100 kbp, which span repetitive regions and allow for the description of complete genetic structures [65,66,67]. This makes third-generation platforms particularly valuable for linking ARGs to MGEs and for studying the functional potential of microbial communities at a broader level. Additionally, real-time sequencing and data generation makes these technologies emerging and convenient tools for rapid diagnostics and AMR detection, allowing for on-site analysis in remote or resource-limited settings. Real-time genomic surveillance is increasingly integrated into hospital settings for real-time tracking of e.g., resistant pathogens, upcoming AMR threats, latent ARGs starting to confer resistance to last-resort antibiotics, and other critical issues that call for immediate action [68,69,70].

However, third-generation platforms often come with higher sequencing costs, higher quality input requirements, and higher computational demands, as well as higher error rates in raw sequences compared to second-generation platforms [60,71]. Despite ONT continuously becoming more accurate, combining the strengths of short-read and long-read sequencing platforms can still offer some advantages for generating more complete and precise genome assemblies and for bridging the gap between taxonomic and functional analyses [72,73].

Compared to traditional culture-based methods, metagenomics combined with advanced bioinformatic pipelines enables the study of both culturable and non-culturable or rare bacteria by eliminating the need for isolation where only a small fraction of environmental bacteria can be cultured [74,75]. This approach provides insights into a broader diversity of microorganisms, giving a more comprehensive understanding of how ARGs are distributed and transferred across different species and diverse ecosystems [76]. Shotgun metagenomics is particularly effective at identifying MGEs, the facilitators of horizontal transfer of ARGs, thus offering a more complete picture of AMR spread. Additionally, it allows for the discovery of novel ARGs within clinical, environmental, and agricultural samples. This makes metagenomics a powerful tool for monitoring a much larger fraction of the pangenome [77].

Altogether, metagenomics is considered a transformative tool that provides a more holistic approach to screening microbial and genetic compartments in ways inaccessible with traditional methods. Metagenomics is invaluable in expanding our current effort to detect AMR dynamics, and in providing early warnings about future threats, supporting a proactive approach to manage AMR. Consequently, some existing public health programs, like NARMS [78] and GLASS [79], have, despite primarily relying on traditional cultivation-based AST methods, started to explore genomic approaches, including metagenomics, for improved AMR surveillance.

### Addressing Limitations and Challenges in Applying Metagenomics in AMR Surveillance

While metagenomics is a promising tool for AMR surveillance, it also faces several challenges and limitations. The accuracy of ARG prevalence estimations based on metagenomic data is, like PCR-based methods, constrained by the limitations of existing ARG databases. We refer to Hendriksen et al. (2019) for a thorough description of in silico AMR detection resources [80]. Numerous and comprehensive ARG databases exist, but they are not complete nor perfect.

An important, often overlooked, concern includes the actual definition of ARGs. Mutations of transporters, regulators, or antibiotic targets can confer antibiotic resistance by limiting uptake or impairing the action of an antibiotic but are not ARGs per se [81]. Conversely, annotated ARGs predicted based on sequence homology do not always confer phenotypic resistance [82,83]. When putative ARGs are decontextualized and cloned onto expression vectors for AMR screening, the results do not necessarily reflect the wildtype function of these genes [84], and hence annotation based on this type of experiments can be problematic but remains abundant in ARG databases [81]. It is also relevant to distinguish between detoxification genes encoded by antibiotic producing microorganisms and genes acquired through HGT as accessory traits in response to antibiotic exposure [81]. Some genes classified as ARGs, like tightly regulated multidrug efflux pumps or β-lactamases, may contribute to the resistant phenotype of a given microorganism, but originally, they evolved to serve basic functions of the bacteria’s physiology and not to provide resistance (native). It is debatable to which degree such genes in their native state pose a risk to human health. Even though putative ARGs may hypothetically transiently confer AMR [85], their inclusion may inflate ARG estimations and lead to biased conclusions of widespread or emerging resistance. Conversely, mutations in transcriptional regulators or the mobilization of putative ARGs may in turn render them highly relevant. Incorporation onto a transposon or integron with an internal promoter or subsequent transfer to conjugative elements or high copy number plasmids can result in highly elevated expression and increases the risk of dispersal [85]. Such events can be induced by antibiotic exposure and the now decontextualized ARGs are likely to pose a higher risk to human health. One example being ESBLs [86]. Hence, it is beneficial to consider the genetic context when evaluating ARG predictions [81,87]. Indeed, studies show that sequence-based detection of ARGs, like ESBL in certain *Enterobacteriaceae* or *mecA* in *S. aureus*, is a more accurate prediction of treatment outcome than AST [34].

Compared to PCR-based methods, shotgun sequencing has the advantage of including information on the genetic context of predicted ARGs and their association with MGEs, which may inform on the likelihood of conferring actual resistance and the risk of spread.

The use of metagenomics in AMR risk assessment is further complicated by user bias introduced via the thresholds chosen for ARG prediction, which if too loose can include false positives leading to overestimations and if too stringent may fail to identify actual ARGs with low sequence similarity to reference ARGs, thus underestimating the presence and potential mobilization [88]. The discrepancy reported between in silico ARG prediction and culture-based methods depends on the study organisms [84]. Metagenomic ARG predictions are more accurate when based on curated ARG databases for well-characterized clinical isolates but may be problematic when large unspecific ARG databases are applied to environmental samples or less characterized species [84]. Another limitation of metagenomics and other culture-independent methods includes the inability to distinguish between viable and non-viable bacteria, which limits its relevance in some clinical contexts. Moreover, metagenomics does not provide quantitative AMR data, such as MICs, which is required for clinical definition of AMR and essential for therapeutic purposes. Thus, while powerful, metagenomics may not fully replace traditional AMR detection methods.

To exploit the full potential of metagenomics for AMR risk assessment, we need well-curated ARG databases. Putative ARGs must not only be assessed based on expression vector assays or solely on sequence homology. We need ground rules for relevant homology thresholds and clear definitions of ARGs pertinent to their genetic context. The use of ranking systems, which divide ARGs according to the associated health risk based on their enrichment, mobility, and the host pathogenicity, as in the omics-based framework proposed by Zhang et al. (2021) [87], could improve the general understanding and handling of AMR threats leading to more informed decision making and better allocation of resources. Novel putative ARGs should ideally be validated in phenotypic studies at clinically relevant concentrations of antibiotics, but such work is exceedingly laborious and not always possible. However, we may have reached a point where technological advances and data availability concurring with reducing costs have made it beneficial to move beyond gene-centric assessment of AMR and adopt a more holistic approach that integrates genomics, transcriptomics, proteomics, and metabolomics. This shift, potentially further enhanced by novel machine/deep learning and AI-driven prediction tools, holds great promise for advancing AMR screening, surveillance, and stewardship [89,90]. However, challenges still remain, one being global inequality. AI algorithms build on training sets and tend to fail when applied to epidemiologically distinct regions. This is problematic as AI development primarily occurs in high income countries, whereas, as mentioned previously, low- and middle-income countries have a higher burden of AMR but lack high-impact data [89]. For a thorough review of the potential of AI and machine learning for AMR mitigation through improved data analysis, predictive modeling, surveillance, and clinical support systems, as well as a discussion of challenges, research gaps and future directions, we refer to Bilal et al. (2025) [90].

## 4. Overview of MGEs and Their Impact on Occurrence and Spread of AMR

**Figure 1:**
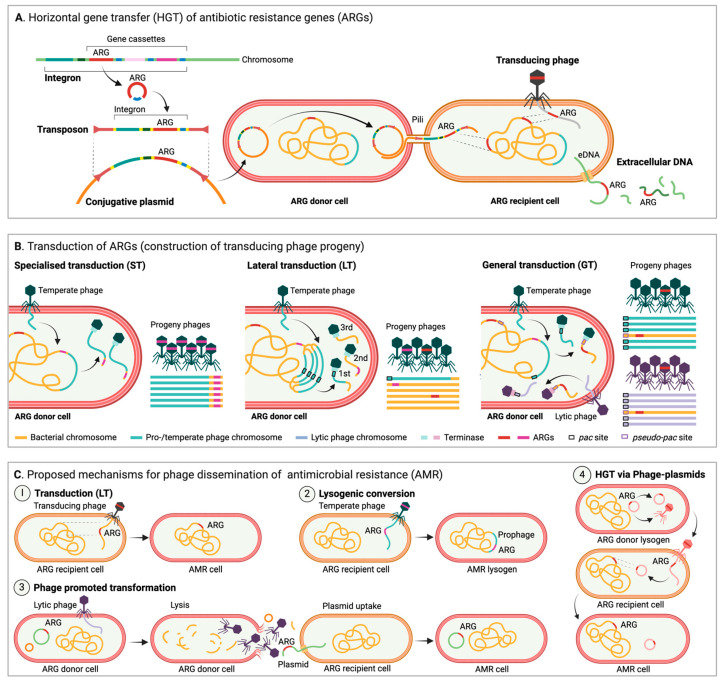
(**A**) Horizontal gene transfer (HGT) of antibiotic resistance genes (ARGs). Conjugation: ARGs can be mobilized via integrons and/or transposons onto plasmids, which can then spread via conjugation. Transduction: transducing phages can deliver ARGs acquired from the DNA of their previous host. Transformation: uptake of extracellular DNA (eDNA) encoding ARGs. (**B**) Transduction of ARGs (construction of transducing phage progeny). Specialized transduction (ST) of ARGs can only occur if ARGs are encoded in very close proximity to a transducing prophage, which inadvertently includes the ARG during excision and then packaging. Lateral transduction (LT) is performed by temperate phages with *pac* site headful packaging, which initiate replication and packaging prior to excision. The terminase packs successive headful fractions of DNA, and as a result only the first virion contains phage DNA, whereas following virions are packed with large, concatenated fractions of the host DNA, potentially including ARGs. Generalized transduction (GT) is performed by both lytic and temperate phages with *pac* site headful packaging. If an ARG is encoded near a *pseudo-pac* site, it can be encapsulated and transferred. (**C**) Proposed mechanisms for phage dissemination of antimicrobial resistance (AMR). Phages can disperse AMR via (1) transduction and hypothetically also via (2) lysogenic conversion (a prophage encoded and expressed ARG). Phages are additionally suggested to (3) promote transformation by facilitating the release of intact plasmids potentially carrying ARGs. Finally, (4) phage-plasmids are more plastid than traditional phages and can acquire ARGs via transposable elements, which they can disperse in their phage state and pass on via e.g., recombination. Figure created in Biorender.

The dynamics of ARG spread across animal, human and environmental sectors are crucial for understanding the evolution of AMR and for predicting the emergence and dissemination of resistant pathogens. Molecular analyses have revealed that widespread MDR has commonly emerged by the rapid acquisition of preexisting, or newly mutated, genetic AMR determinants, followed by their amplification in response to selection [7,10,91]. MGEs are mainly responsible for facilitating intracellular DNA mobility (e.g., transfer of genetic traits from the chromosome to MGEs), as well as intercellular transfer of genetic information between bacterial cells and across species [12,14,92]. ARGs are often encoded as accessory elements on MGEs [93], such as integrons, transposons, plasmids, or phages (Figure 1A). Consequently, MGEs play a central role as vectors in promoting the capture, accumulation, and dissemination of resistance traits across bacterial species and environments in response to selection.

In brief, integrons contain a site-specific recombination system and are notable for their ability to capture, integrate, and modulate the expression of gene cassettes, often including ARGs, which makes them important drivers in the rapid spread of AMR traits [94,95]. Integrons are not self-mobile, but they often hitchhike on other MGEs like plasmids or transposons, allowing their spread across bacterial populations (Figure 1A) [95]. In contrast, transposons, also commonly known as “jumping genes”, can move from one location to another within a genome, or between genomes, via transposase activity [96,97]. Besides carrying transposase enzymes facilitating the transposition process, transposons often encode accessory genes that confer AMR. As they can integrate into various genetic environments, such as chromosomal DNA, plasmids, or other MGEs, transposons typically contribute significantly to the adaptability and evolution of bacterial populations (Figure 1A) [12].

Unlike transposons and integrons, plasmids replicate autonomously of the host chromosome and are easily transferable between bacteria via HGT mechanisms [12,98,99], in particular conjugation (Figure 1A), which is considered to be the most frequent type of HGT (as compared to transformation and transduction) in natural environments [100,101]. As plasmids often carry genes that confer advantageous traits, including ARGs, virulence factors, or metabolic capabilities, they are often recognized as some of the most central players in the dissemination of AMR [102]. Finally, phages are known to play a dual role in bacterial evolution due to their ability to infect bacteria. Infection typically occurs either through a lytic cycle, in which phages replicate within and kill their bacterial hosts [103], or through a lysogenic cycle where they as temperate phages integrate their genomes into the host chromosome becoming prophages that remain dormant until triggered [104,105]. Typically, phages contribute to the spread of AMR through transduction, where bacterial genes are packaged into phage particles enabling their mobility and transfer to other bacteria (Figure 1B).

Together, these MGEs form a dynamic network that contributes to the genetic diversity and evolution of bacterial hosts and, notably, allows the acquisition, movement, and sharing of genetic resistance determinants across different microbial environments. Importantly, pathogenic bacteria can easily obtain resistance via HGT of MGEs carrying AMR determinants, clearly illustrating the ability of HGT to facilitate and speed-up the spread of AMR from commensal and environmental bacteria to pathogens in clinical environments.

### The Role of Phages, an Underestimated Driver of AMR Transmission?

The importance of phages as mediators of ARG transfer through transduction (Figure 1B) is not yet fully explored. Most research on HGT of AMR focuses on conjugation and transformation [106]. But several studies suggest that generalized transduction (GT) (Figure 1B), the perceived “accidental” packaging and transfer of “random” host DNA by phages with *pac* site driven encapsidation, can be induced by antibiotic exposure and can facilitate the spread of ARGs within pathogens, including *Acinetobacter baumannii* [107], *E. coli* [108], and *Salmonella* [109,110]. GT is also a major contributor to the spread of ARGs in *S. aureus* and consequently responsible for the success of this pathogen [108,111,112,113]. It has been proposed that GT is indeed an intrinsic part of phage biology that benefits temperate phages and promotes the survival of both lysogen and phage in changing environments and may not be as random and accidental as previously anticipated, as *pac*-like sequences (*pseudo-pac* sites) in the host chromosome can act as preferential hotspots for transduction for, e.g., Salmonella phage P22 [111,114], and as ARG packaging by T7-like phages infecting MDR *E. coli* appears to be preferential rather than random [115].

Traditionally, two types of transduction have been recognized, GT as described above and specialized transduction (ST) (Figure 1B), which involves only small fractions of host DNA adjacent to the prophage attachment site (e.g., the *gal* or *bio* operons by phage λ, [116]) transferred due to irregular prophage excision. ST may be responsible for temperate phages acquiring ARGs [117] but is unlikely to be a major contributor to ARG mobilization. However, a third and more potent type of transduction has been identified in *S. aureus*, *P. aeruginosa*, and *Salmonella* [118,119]. Lateral transduction (LT) (Figure 1B,C.1) is, like GT, performed by temperate phages with *pac* site headful packaging, but here late excision and in situ replication ensure high frequency transfer of large proximate fractions of the host chromosome of up to seven headfuls spanning up to several hundred kbp [111,118], potentially including ARGs [114,120]. LT is a powerful mechanism that can facilitate the transfer of “immobile” core genes at high frequencies up to 1000-fold higher than in GT [118] at times exceeding that of established MGEs [120].

Unlike as for traditional MGEs, transduction mobility is not coupled to the genetic element but by proximity to prophages [121]. It has been verified that transduction can make sensitive pathogens resistant towards multiple antibiotics, i.e., *S. epidermidis* to streptomycin, tetracycline, and chloramphenicol [122], and even spread resistance to tetracycline and gentamycin across species of *Enterococcus* [123]. This indicates that phages could be important mediators of AMR for select bacteria through transduction, but dispersal via transduction is narrowed by the host specificity of the phage, which is often constricted to very few species or even strains (though co-infection with unrelated phages and the risk of chimeras open for a broader range of spread). Furthermore, in both GT and LT the encapsidated bacterial genomic fragments will often lack the phage genes required to prevent degradation from the host defense system [124], suggesting that transformation and conjugation are likely more important for the wider spread of ARGs [119]. However, further studies are required to determine the range and frequency of transduction of AMR in non-laboratory settings.

Phages have also been suggested to encode and spread ARGs through phage–host interactions like lysogenic conversion (Figure 1C.2) [82], but the concept of phages as reservoirs for ARGs is controversial and remains a subject of debate [125,126,127]. In line with Pfeifer et al. (2022), and to the best of our knowledge, a natural non-defective phage encoding innate bona fide ARGs capable of providing AMR through lysogenic conversion in repeated infection cycles has still not been described [128], and functional phage-encoded ARGs may indeed be rare [126]. In a recent study analyzing 38,861 phage genomes, Kant et al. (2024) identified only 314 ARGs in 182 phages, and when screening several environmental viral and metagenomic databases they found that temperate phages and prophages (2.15% and 0.66%, respectively) were most likely to carry ARGs and had likely acquired these through ST [117]. These findings ascertain phages as contributors to ARG dispersal but also support the notion that the scarcity of phage-encoded ARGs combined with transfer bottlenecks limits their role. Conversely, another recent study by Liao et al. (2024) on 38,605 bacterial genomes, 1435 metagenomes, and 1186 meta-transcriptomes describes a significant increase in the abundance, diversity, and activity of prophage-encoded ARGs correlating with the likelihood of anthropogenic antibiotic exposure and suggests that “*prophages serve as globally important, hidden reservoirs for ARGs*” [82]. Liao et al. (2024) found ARGs in almost half of the identified prophages, but practical constraints limited the experimental validation (by decontextualization and expression in *E. coli* DH5ɑ) to only six ARGs, of which just three increased the level of resistance [82], suggesting that caution should be taken when interpreting ARG predictions. These two studies both assessed multiple large genomic and metagenomic resources, and both used the CARD database [129] to identify phage ARGs, but whereas Liao et al. (2024) used the Resistance Gene Identifier with “strict” parameters (allowing detection of variants) [82], Kant et al. (2024) performed standalone BLASTN with a stringent 80% threshold for both coverage and identity [117], which may explain some of the discrepancy and highlights the need for a more uniform approach.

Diverse clinically relevant and verified ARGs have been observed in phage-related MGEs, such as phage-plasmids, which are abundant in pathogens [128]. Occasional encapsidation enables these phage-plasmid-encoded ARGs to reach bacteria distant in space and time from the original host (Figure 1C.4) [128]. Phage-plasmids encode ARGs, which appear to have been co-translocated with transposable elements, often in class I integrons, and hence phage-plasmids may also be important for the transfer of genes, including ARGs, across MGEs [128,130]. In addition, so-called “super-spreader” phages have been suggested to promote substantial transformation of plasmids (Figure 1C.3), thereby indirectly facilitating dispersal of ARGs [131]. To sum up, the role of phages, both direct and indirect, in the mobilization of ARGs is highly complex (Figure 1C). More studies are required to fully understand how and to what degree phages spread ARGs in diverse environmental settings, and the many conflicting indications underline the need for a standardized method for in silico ARG identification and for experimental validation.

## 5. Integration of MGE-Based Metagenomic Data for Improved AMR Surveillance

As different types of MGEs interact with each other to recruit and disseminate accessory genes, such as ARGs, rapidly throughout complex microbial populations, the ability to identify, characterize and monitor the occurrence of MGEs becomes essential in order to elucidate AMR epidemiology [132,133,134]. Recent advances in metagenome sequencing and bioinformatics have helped us to significantly improve the development of tools aiming to provide a more detailed and comprehensive tracking of MGEs to increase the surveillance effort on these in relation to their anticipated impact on occurrence and propagation of AMR across different environmental settings. Also, the increased availability of metagenomic sequencing data from diverse ecosystems has allowed us to study the distribution and diversity of MGEs on a global scale [135,136].

To date, several bioinformatic tools have been developed that can identify and analyze a wide range of MGEs associated with AMR in bacterial genomes and metagenomic datasets. Here, some of the most recent approaches are highlighted, including their advantages and limitations (Table 1).

Many of the databases and web-based tools listed in Table 1 represent approaches perfectly suitable for detecting and classifying various types of MGEs in different environmental samples, and some tools are even capable of processing complex metagenomic datasets. Several of the listed tools, such as MobileElementFinder [139] and BacAnt [152], offer the possibility of associating the identified MGEs to specific genes, such as ARGs, thereby providing the opportunity of potential AMR gene linkage. Interestingly, some plasmid classification tools like MOB-suite [144] and OriTfinder [140] offer mobility predictions, which are critical for understanding AMR dissemination dynamics. On that same note, phage identification and annotation tools like PHASTER [148], VirSorter2 [151], and VIBRANT [150] are all to some extent able to detect phages with the ability to integrate within bacterial genomes as prophages, potentially aiding in the study of tracking phage-mediated AMR dissemination.

In general, however, many of the listed tools to some extent suffer from the same limitations and pitfalls that limit their accuracy. Despite allowing the differentiation of chromosomal DNA from phages, plasmids, and other MGEs, their discovery and characterization of MGEs remains partially constrained by existing, and often incomplete, reference databases and training sets that are based on known MGEs. Hence, they include a risk of missing novel, divergent and unexpected MGEs. Importantly, one should keep in mind that the success of detecting and describing emerging MGEs to a large degree depends on the cut-off thresholds chosen to screen for genetic similarity to known MGEs, which all-together makes unbiased detection of unknown MGEs a major challenge [153].

Many existing tools require bioinformatics expertise (i.e., lack user-friendly mode of operation) and can be biased towards types of bacteria extensively studied, such as pathogens [154,155], whereas less-studied bacteria become underrepresented. Moreover, several of the listed tools like MobileElementFinder [139], ICEBERG 2.0 [141], IntegronFinder 2.0 [142], ISEScan [143], and PHASTER [148] require high-quality sequencing data for optimal results and hence might not work well (i.e., lead to longer processing times and/or reduced performance) when applied to more complex metagenomic datasets containing chimeric or incomplete/fragmented sequences [156]. Finally, many available tools are specialized for specific types of known MGEs, and no single solutions seem available to detect a more comprehensive view of multiple types of MGEs. Hence, chosen methods always need to be evaluated beforehand and typically used in combination to provide the best solution for the given case [76,157].

To date, tools based on machine learning, especially deep learning, have the emerging potential to address several of the limitations associated with traditional bioinformatic tools through their ability to recognize patterns in metagenomic data beyond explicit database matches [158]. Many machine learning models, like those used in geNomad [136], are continuously trained on large and new datasets, which allows them to predict novel MGEs and ARG mobility based on sequence features, even when no direct database match exists. This allows them to adapt to emerging MGEs more rapidly than when database-dependent tools are applied. On the same note, trained machine learning models have the potential to process large and complex datasets more efficiently than traditional rule-based algorithms, and they can analyze multiple types of MGEs in a more comprehensive framework, hence reducing the need for combining several specialized databases and tools [159,160,161].

With that said, the effectiveness of current machine learning-based tools depends on the quality and diversity of the training data, and biases in training sets could potentially limit their performance [162,163]. Also, training and running deep learning models are still at a level that can be computationally expensive and exhausting, which should be taken into consideration. Despite the potential of deep learning to unify many functionalities, they are still unlikely to completely replace traditional tools, and the best results within MGE and AMR research might often come from combining them with traditional tools and databases to exploit the complementary strengths.

However, given the diverse range of bioinformatic tools available for AMR surveillance in metagenomic datasets, tool selection should be guided by the specific research objective, data type, and quality. As a recommendation, traditional database-dependent tools such as PlasmidFinder [147], MobileElementFinder [139], and IntegronFinder 2.0 [142] remain highly effective for detecting known ARGs and specific types of MGEs in well-characterized datasets. In contrast, machine learning-based tools like geNomad [136] offer the potential of identifying novel MGEs and predicting functional AMR dynamics. For large-scale metagenomic studies, tools such as MOB-suite [144], MGEfinder [138], and VIBRANT [150] are well suited for tracking MGEs and their association with ARGs, whereas PHASTER [148] and VirSorter2 [151] are valuable for exploring phage-mediated AMR transmission. As the landscape of bioinformatic tools is constantly evolving, these recommendations should be seen as flexible guidelines rather than definite rules.

Interestingly, as machine learning approaches are capable of integrating diverse types of data (e.g., sequence similarity, genomic signatures, CRISPR spacers), they can be useful in improving the accuracy of host-MGE association predictions from metagenomic data, which is often recognized as a significant challenge [164]. These approaches are, therefore, highly valuable as establishing links between MGEs and their host organisms could enrich our current understanding of AMR dynamics and help to predict future resistance threats and guide surveillance efforts towards high-risk hosts and/or environments. Knowing the host not only allows researchers to identify which species act as reservoirs for resistance genes, but some MGEs exhibit preferences for certain bacterial hosts [165,166], which could provide insight into how MGE-carried ARGs move within populations or between environments (e.g., hospital-acquired infection vs. community-acquired infections).

Here, geNomad [136] integrates machine learning approaches to classify MGEs and predict their potential host range, but also the bioinformatic toolbox MGEfinder [138] has the ability to pinpoint MGE integration sites, emphasizing to identify the interaction of MGEs with their bacterial hosts, distinguishing it from broader tools that detect MGEs without linking them to their genomic context. Other tools have recently been developed to predict MGE-host linkage, such as PlasmidHostFinder that utilizes random forest-based machine learning models to predict the host range of plasmids at several bacterial taxonomic levels [167].

On that note, emerging trends also involve the prediction of host-MGE associations by analyzing DNA methylation patterns and other epigenetic modifications that are often host-specific. MGEs, such as plasmids and phages, can acquire epigenetic signatures from their host organisms during replication. By comparing these epigenetic markers, such as methylation profiles detected through single-molecule sequencing technologies like PacBio or ONT, the likely host of a specific MGE can be identified [168].

Overall, combining huge metagenomic datasets gathered across complex MGE-harboring microbial communities with machine learning-based approaches might hold the key to efficiently increase our current understanding of the ecology and environments in which MGEs are disseminated. This not only provides insights into how ARGs as accessory traits are transferred between bacterial species and environmental settings but helps to fill crucial knowledge gaps about the intricate relationship between MGEs and their bacterial hosts. This in turn allows us to better predict and manage emergent resistance trends and identify hotspots of AMR spread, hence improving our current AMR surveillance strategies.

## 6. Phage Therapy, a Renewed Approach to Treat (AMR) Bacterial Infections?

Phage therapy (PT), the use of primarily lytic phages to treat bacterial infections, including those caused by AMR pathogens, is being revisited. Accordingly, surveillance of phage–bacteria interactions and their impact on AMR development is becoming increasingly important.

PT development and implementation is complicated by the host specificity of phages. This is addressed by two types of approaches: (1) broad-spectrum phage cocktails targeting bacterial pathogens causing a specific disease or (2) the compilation of phage biobanks to enable a swift screening for suitable phage(s) for a specific patient (personalized PT). Both types are laborious and expensive and involve a risk of phage resistance development, which, especially when using cocktails, may also affect non-disease-causing bacteria. Bacteria encode a wide range of anti-phage defense systems [124]. However, when exposed to a high number of lytic phages, as in a phage treatment scenario, a common mode of phage resistance is (transient) downregulation or occlusion of phage receptors. Phages have diverse receptors, but they include virulence and resistance factors such as OMP, LPS, capsules, motility factors, and efflux pumps [169,170]. Therefore, phage exposure can render pathogens less virulent and more susceptible to antibiotics and in some cases even to previously ineffective antibiotics. Even though this effect is restrained by specific phage–host interactions, type of antibiotic, genotypes, and environment, e.g., biofilm formation [170], it can improve treatment outcomes and also has important implications for AMR progression [171].

Besides the risk of phage resistance potentially rendering expensive and, in some cases, last resort treatments ineffective [172], clinical application of PT also faces other challenges. Phages are generally considered safe for human treatment, granted production ensures removal of residual bacterial host toxins, endotoxins, and superantigens [173]. However, phage-induced immune responses are underexplored and can affect phage bioavailability and consequently reduce the therapeutic potential especially in cases of prolonged treatment with the same phages [173], complicating PT development. In addition, Big Pharma is abandoning antimicrobial research and development, leaving the market to relatively smaller companies [174]. Consequently, private investment in PT is impeded by a commercial pressure to obtain high profit margins, which favors investments in more lucrative therapies [174]. Furthermore, the host-specificity and viral nature of phages makes them difficult to fit in traditional business models [174]. Another major hurdle is regulatory barriers. In the present day, there is no legal framework in place for clinical implementation of PT in neither the US nor the EU, and no phage medicinal drugs have been approved for human use [175,176]. Both the European Medicines Agency (EMA) and the US Food and Drug Administration (FDA) are, however, working towards this [175,176]. Meanwhile, many countries, including Georgia, Poland, Belgium, France, Sweden, Denmark, the United Kingdom, Australia, and the US, have enabled the clinical use of phages as magistral preparations (or corresponding) for compassionate use [171,174,176].

As a result of these challenges, current western use of PT is mainly for compassionate use in difficult-to-treat cases, often involving AMR infections. Phage treatments are usually administered in combination with antibiotics, which tend to increase the probability of bacterial eradication [170,172]. Even though PT use is on the rise globally [171], clinical PT trials, though increasing in numbers, are still limited [171]. A recent analysis of 100 consecutive personalized PT cases across 35 hospitals in 12 countries shows promising results in 77.2% cases with clinical improvement and 61.3% with bacterial eradication. These data could prove very useful for the design of future controlled clinical trials [172], which in turn will aid in drawing more finite conclusions on the efficacy and suitability of PT and to develop standardized and scalable protocols.

The development of progressively powerful technologies like AI and synthetic biology may very well speed up this process by enabling both the selection and modification of natural phages for optimal efficacy or even the design and construction of de novo synthetic phages or phage components tailored for a specific treatment. A solution which could revolutionize PT by enabling on-site timely identification, cell-free production and verification of personalized phage medicines [173,174], reducing the need for time-consuming long-distance phage requests, costly maintenance of large PT biobanks, and the need for antibiotic treatments [173,174]. Furthermore, it would minimize the risk of PT inadvertently contributing to AMR dissemination by guiding the selection and/or design of phages/phage products to avoid unwanted traits potentially resulting in ARG mobilization as described in the section on phages and AMR transmission.

A global One Health implementation of PT as a supplement to conventional antibiotics, in not just medicine but also agriculture, aquaculture, husbandry, and veterinary medicine, would drastically lower the selection pressure for AMR development and consequently reduce the spread of ARGs. But, to fully exploit the potential of phages and avoid unintentional and undesired side effects, we need not only more empirical data but also a globally coordinated approach and the development of shared resources like phage biobanks and access to GMP facilities [171]. Data collection and analysis is the foundation for progressing with PT implementation. By obtaining a better understanding of in vivo efficacy, immune responses and the wider ecological effects of PT, medical personal, researchers and decision makers will be better equipped to direct the use of and investment in PT. Making therapeutic phage monitoring (TPM) an essential element of PT protocols would drastically contribute to the generation and compilation of such data, filling in knowledge gaps and providing the foundation for optimizing and standardizing guidelines and protocols for both treatment and manufacturing [171,177].

## 7. Conclusions

In summary, ongoing challenges associated with combating AMR on a global scale highlight the need of implementing multifaceted approaches in current surveillance strategies. A critical aspect of this effort is the monitoring of MGEs, which play a crucial role in the dissemination of ARGs across diverse microbial populations and ecosystems. The integration of advanced metagenomic sequencing technologies and bioinformatics tools is essential for improving our ability to identify and characterize MGEs, as well as increasing our understanding of their dynamics in various environmental contexts. However, existing bioinformatic pipelines tend to face certain limitations, including dependency on high-quality sequencing data, biases towards well-studied pathogens, lack of user-friendly interfaces and biases in detecting unknown MGEs. Machine learning-based approaches have progressed remarkably in recent years and represent a significant and emerging trend in this area by offering the potential to analyze complex and large (meta)genomic datasets and predict novel MGEs. Continuous refinement of surveillance pipelines based on machine learning will strengthen global efforts to track the occurrence and spread of AMR, ultimately helping to safeguard public health strategies and interventions.

The increase in affordability of sequencing technologies combined with the on-going advancements within machine learning-based AMR prediction make large-scale implementation of metagenomic surveillance in clinical and environmental settings more feasible in the near future. However, we also need to acknowledge that sequencing and computational costs might still act as barriers in resource-limited settings and that further actions are needed with regard to better standardization of bioinformatic pipelines and databases to ensure reliable AMR gene prediction. Also, ethical concerns raise the requirement for frameworks addressing data privacy, sample collection, and reporting guidelines.

The role of phages as mediators of AMR transmission through transduction is increasingly recognized but remains underexplored. In particular, LT, performed by temperate phages with *pac* site headful packing, has the ability to transfer large fractions of the host chromosome, potentially including ARGs. However, transduction of ARGs is limited by the narrow host range of most phages, though certain circumstances might allow for a broader range of spread. Moreover, initial observations of so-called “super-spreader” phages promoting transformation of plasmids suggest an indirect role in the mobilization of ARGs. Nonetheless, the potential role of phages as direct or indirect facilitators of AMR spread remains complex and highly debated, which strongly underlines the need for more experimental validation and research within this area.

Finally, it is worth highlighting PT as a promising supplement to conventional antibiotics. As the global landscape of AMR evolves, the development of personalized PTs, supported by technologies like AI and synthetic biology, could revolutionize treatment protocols. This approach would reduce the current dependency on traditional antibiotics and mitigate the selection pressure that drives AMR. The implementation of PT does, however, face several challenges, including a legal framework for implementation. There is a need for a coordinated global effort to establish shared resources like phage biobanks, access to GMP facilities, and standardized guidelines and protocols for treatment and manufacturing. Recognizing TPM as an essential part of PT could significantly strengthen and speed up any progress within this area and, therefore, represents an important step toward preserving the efficacy of existing antibiotics.

## Figures and Tables

**Table 1:** Overview of selected bioinformatic approaches for identifying and analyzing AMR-associated MGEs.

Name	Type	Description	Strengths	Limitations
MobileGeneticElementDatabase [137]	Varied MGEs	Non-redundant fasta format database with annotation files.	Cross-reference with other databases; links MGEs to broader functional contexts. Includes metadata, e.g., host and environment.	May not always capture the most recently discovered or rare MGEs. Updates and curation frequency vary.
MGEfinder [138]	iMGEs	Identifies iMGEs (primarily IS and transposable elements) and their insertion sites using short reads.	Precise annotations of MGE-host interactions (insight into HGT events).	Highly fragmented assemblies may reduce accuracy. Relies on known sequence features, might miss novel elements.
MobileElementFinder [139]	iMGEs and conjugative Tns	Webserver; detects iMGEs (MITEs, ISs, ComTns, PCTs, Tns and conjugative Tns (ICEs, IMEs and CIMEs)), annotates in relation to AMR.	User-friendly and highly specific detection of iMGEs in bacterial genomes based on curated databases and sequence motifs. Links MGEs to ARGs.	May not perform as well on high complexity metagenomic datasets. Relies on known databases; limited detection of novel iMGEs. Requires high-quality assemblies.
OriTfinder [140]	ICEs, conjugative plasmids, AMR plasmids.	Identifies and annotates oriT regions in bacterial DNA sequences of conjugative plasmids and ICEs.	Insight into HGT/mobility of plasmids and their role in spread ARGs. Integrates with multiple databases to predict plasmid transfer genes.	Focused on oriT and relaxase genes. No comprehensive overview of MGEs. Limited to known oriT and relaxase sequences, potentially missing novel elements.
geNomad [136]	MGEs, specifically plasmids, virus and prophages.	Integrates ML approaches to identify and classify MGEs in meta-/genomic datasets, based on nucleotide composition.	Advanced algorithms and ML enable reliable classification and accurate detection of varied MGEs, incl. atypical features. Prediction of bacterial host(s). Suitable for large metagenomic datasets.	Accuracy depends on training-dataset quality; novel/rare MGEs might be missed. Requires significant computational power for large-scale analyses. Host range prediction is probabilistic and might not be accurate for MGEs with unknown associations.
ICEBERG 2.0 [141]	ICEs	Specialized database for identification, classification and annotation of ICEs in bacterial genomes.	Tools to identify ICEs based on integrase genes, attachment sites, and conjugative machinery. Facilitates comparative analysis by linking ICEs to host genomes.	Limited to identifying known ICE features; novel ICEs may not be detected. Requires high-quality genome assemblies for accurate identification.
IntegronFinder 2.0 [142]	Integrons	Identifies and annotates integrons and their associated gene cassettes in bacterial meta-/genomes.	Supports the detection of atypical integrons. Can process large-scale datasets, including draft genomes and metagenomes. Visualization of integron structures.	Relies on sequence quality; low quality may result in incomplete detection. Reduced speed at large datasets. Focused on integrons and their components.
ISEScan [143]	IS elements	Based on profile hidden Markov models constructed from manually curated IS elements. Identification of ISs in bacterial meta-/genomes.	Capable of identifying a wide range of IS families. Provides detailed annotations of IS elements, including their functional domains.	Requires well-assembled sequences for optimal performance. Limited to ISs; does not detect other MGEs.
MOB-suite [144]	Plasmids	Characterization, clustering and replicon typing of plasmids from WGS draft assemblies.	Prediction of plasmid replication host range and mobility potential. Incorporates up-to-date plasmid sequence data. Links ARGs to specific plasmids, relevant in the context of AMR dissemination.	Performance is limited by the reference database, with reduced accuracy for poorly characterized or novel plasmids. Computationally demanding for large datasets, requires some bioinformatics expertise.
PlasFlow [145]	Plasmids	Neural network-based tool for predicting and classifying plasmid sequences in metagenomic contigs from environmental samples.	High accuracy in distinguishing plasmid-derived sequences from chromosomal DNA. Suitable for large-scale metagenomic datasets.	Supervised ML approach, limited by training test; may not classify novel sequences well. Similar backbone elements and short sequences might complicate plasmid classifications.
PlasClass [146]	Plasmids	Algorithm (ML) that classifies contigs in meta-/genomic assemblies as plasmid or chromosomal DNA.	Useful for metagenomic datasets with short, fragmented or incomplete sequences. Can identify plasmid sequences even without known replicons.	Accuracy may vary with highly fragmented assemblies. Novel plasmid types might not be well-represented in training datasets.
PlasmidFinder [147]	Plasmids	Web-based; detects and identifies plasmid replicons in bacterial genomes.	Highly accurate identification of known plasmid replicons including Inc group assignment. Immediate plasmid classification. For WGS, accepts raw sequence data.	Relies on known plasmid replicon sequences; does not detect novel plasmid types. Does not provide detailed information on plasmid structure or the specific genes carried on the plasmid.
PHASTER [148]	Prophages	Identifies and annotates prophage sequences within bacterial genomes and plasmids.	User-friendly web interface. Detailed visual output of prophage regions. Continuously updated database improves accuracy. Suitable for complete and draft bacterial genomes (meta-/genomic).	Performance declines with highly fragmented genomes/incomplete assemblies. Longer processing times for large datasets (faster version; PHASTEST [149] for metagenomic/fragmented datasets, cursory annotation).
VIBRANT [150]	Phages, prophages	ML; identification and functional annotation of phages in metagenome data, especially prophages.	Accurate prophage detection, activity assessment and infection mechanism. Interactive outputs, including detailed annotations and visualization. Can identify a wide range of phages, including novel ones.	Computationally demanding for large datasets. Needs command-line expertise for local installation.
VirSorter2 [151]	Phages, prophages	DNA and RNA virus identification and multi-classifier tool, differentiates between prophages and free viruses in meta-/genomic datasets.	Detects diverse viral sequences (complete and partial), including novel viruses and prophages, using ML and curated regularly updated databases. Can process large-scale metagenomic datasets.	May classify some host-derived sequences as viral, leading to false positives. Accuracy depends on the completeness of viral reference databases and may miss highly divergent viruses. Computationally demanding for large datasets.
BacAnt [152]	ARGs, integrons, transposable elements	Web-based tool tailored for predicting ARGs, integrons and transposable elements in genome sequences.	Provides detailed functional annotations of ARGs, integrons and transposable elements in a single step. Generates Genbank files for comparative genomic analysis.	Limited database coverage for rare or emerging genes; novel elements may be missed.

**Abbreviations:** machine learning (ML); mobile genetic elements (MGEs); integrative MGEs (iMGEs); integrative conjugative elements (ICEs); miniature inverted repeats (MITEs); insertion sequences (ISs); composite transposons (ComTns); pseudo-composite transposons (PCTs); unit transposons (Tns); integrative mobilizable elements (IMEs); cis-mobilizable elements (CIMEs); whole-genome sequencing (WGS); antimicrobial resistance genes (ARGs); antimicrobial resistance (AMR); horizontal gene transfer (HGT); origin of transfer (oriT).

## Data Availability

Not applicable, no new data was generated for this review.
